# Therapeutic implications of intratumor heterogeneity for *TP53* mutational status in Burkitt lymphoma

**DOI:** 10.1186/s40164-015-0019-9

**Published:** 2015-08-27

**Authors:** Enrico Derenzini, Ilaria Iacobucci, Claudio Agostinelli, Enrica Imbrogno, Clelia Tiziana Storlazzi, Alberto L`Abbate, Beatrice Casadei, Anna Ferrari, Andrea Ghelli Luserna Di Rora`, Giovanni Martinelli, Stefano Pileri, Pier Luigi Zinzani

**Affiliations:** Department of Experimental, Diagnostic and Specialty Medicine, DIMES, Institute of Hematology and Medical Oncology L.A. Seragnoli, University of Bologna, Via Massarenti 9, 40138 Bologna, Italy; Hematopathology Unit, Department of Experimental, Diagnostic and Specialty Medicine, DIMES, University of Bologna, Bologna, Italy; Department of Biology, University of Bari “Aldo Moro”, Bari, Italy

**Keywords:** Burkitt lymphoma, Intra-tumor heterogeneity, Genomic instability, CHK1, γ-H2AX, MYC

## Abstract

Therapeutic implications of intra-tumor heterogeneity are still undefined. In this study we report a genetic and functional analysis aimed at defining the mechanisms of chemoresistance in a 43-year old woman affected by stage IVB Burkitt lymphoma with bulky abdominal masses and peritoneal effusion. The patient, despite a transient initial response to chemotherapy with reduction of the bulky masses, rapidly progressed and died of her disease. Targeted *TP53* sequencing found that the bulky mass was wild-type whereas peritoneal fluid cells harbored a R282W mutation. Functional studies on *TP53* mutant cells demonstrated an impaired p53-mediated response, resistance to ex vivo doxorubicin administration, overexpression of DNA damage response (DDR) activation markers and high sensitivity to pharmacologic DDR inhibition. These findings suggest that intra-tumor heterogeneity for *TP53* mutational status may occur in *MYC*-driven cancers, and that DDR inhibitors could be effective in targeting hidden *TP53* mutant clones in tumors characterized by genomic instability and prone to intra-tumor heterogeneity.

**Electronic supplementary material:**

The online version of this article (doi:10.1186/s40164-015-0019-9) contains supplementary material, which is available to authorized users.

## Background

Genomic instability, defined as the tendency to acquire DNA damage determining accumulation of genomic alterations over time, is a hallmark of cancer conferring evolutionary advantages, and resulting in resistance to chemotherapy and increased metastatic potential [1]. A common mechanism determining genomic instability in tumors is oncogene-induced replication stress, leading to DNA damage accumulation during the S phase of the cell cycle [2]. Our group and others recently reported that *MYC*-driven cancers such as Burkitt lymphoma (BL) and Diffuse large B-cell lymphoma (DLBCL) overexpress active components of the DNA damage response pathway (DDR) such as checkpoint kinases (CHK1/2), in order to cope with the high levels of replication stress deriving from *MYC* overexpression, and are sensitive to pharmacologic DDR inhibition [3–5]. BL is characterized by a high level of *MYC* expression due to the occurrence of chromosomal translocations which are hallmarks of the disease, and G1/S checkpoint dysfunction with frequent *TP53* mutations (30 % of cases) [6, 7]. *TP53* mutations drive chemoresistance in many different cancers including aggressive B-cell lymphomas [8, 9], and cooperate with MYC by preventing its intrinsic proapoptotic effects and by further increasing genomic instability [10]. Intra-tumor heterogeneity, intended as the occurrence of genomic diversities within the same tumor over space and time, is intimately related to genomic instability, and has been recently unraveled by next generation sequencing (NGS) studies [11]. Nevertheless, its clinical significance and therapeutic implications in aggressive B-cell lymphomas are yet to be elucidated.

In the current study we report a genetic and functional analysis aimed at defining the mechanisms of chemoresistance in a 43-year old woman affected by stage IVB Burkitt lymphoma with bulky abdominal masses and peritoneal effusion. The patient, despite a transient initial response to chemotherapy with reduction of the bulky masses, rapidly progressed and died of her disease. Targeted *TP53* sequencing found that the bulky mass was wild-type (WT) whereas peritoneal fluid cells harbored a R282W mutation, depicting a paradigmatic example of intra-tumor heterogeneity for the *TP53* mutational status at disease onset in BL. Functional studies on the *TP53* mutant clone confirmed an impaired p53-mediated response and resistance to ex vivo doxorubicin administration. Finally, we demonstrated that these cells were characterized by overexpression of markers of genomic instability and DDR pathway activation, and were sensitive to pharmacologic inhibition of CHK kinases.

## Case presentation

**Fig. 1:**
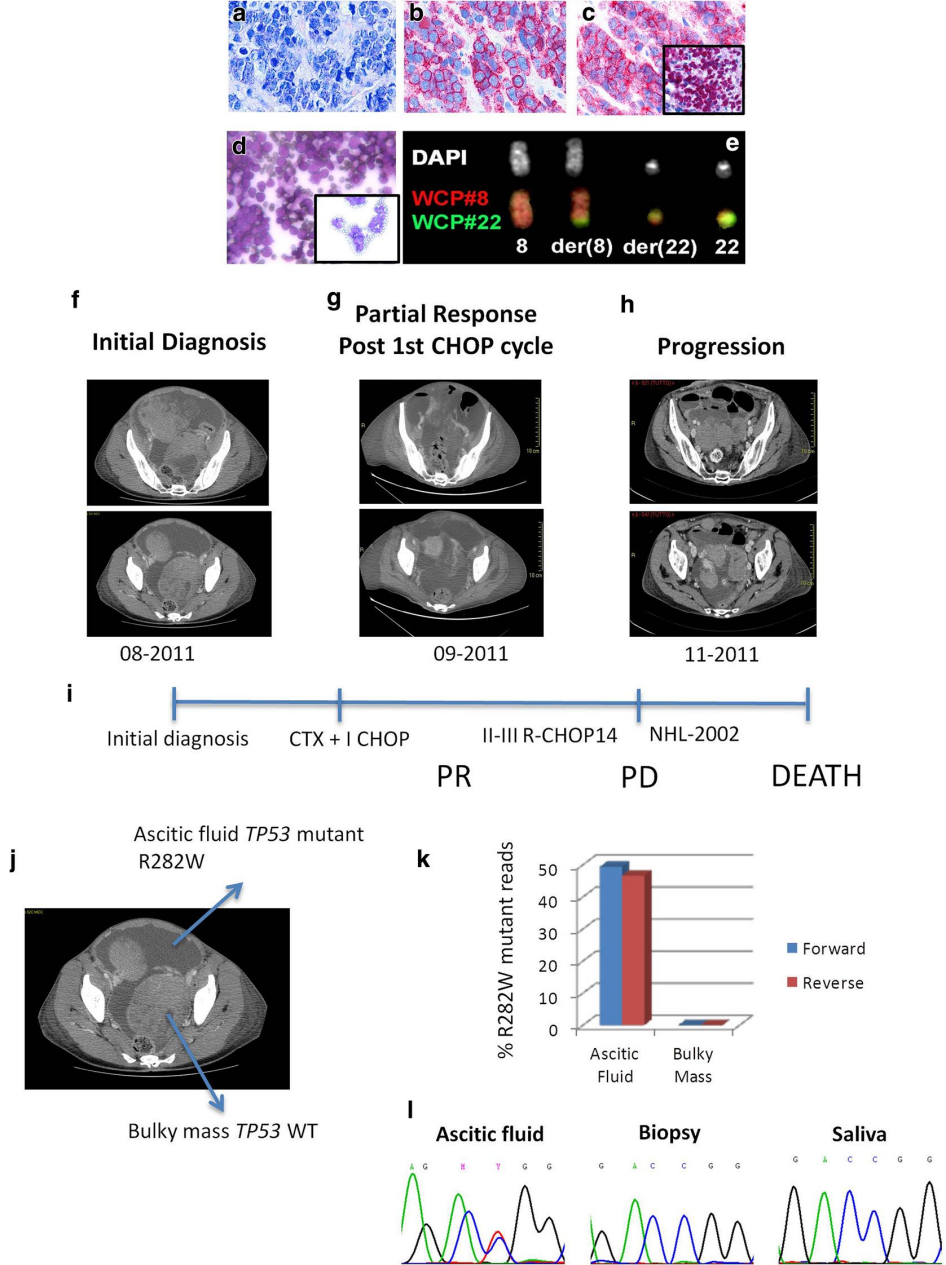
Clinical history, therapeutic interventions and *TP53* sequencing results. **a**–**d** Immunohistochemistry slides showing Burkitt lymphoma medium-sized cells (Giemsa stain) expressing CD20 (**b**) and CD10 (**c**) (×400); c-MYC positivity in the inset (**c**). Peritoneal fluid collected at the moment of initial diagnosis (**d**), showing monomorphic BL cells with frequent mitotic figures. **e** FISH analysis of cells from peritoneal fluid using the Whole Chromosome Painting (WCP) probes of chromosomes 8 and 22, respectively pseudo-colored in *red* and *green*. The results showed the occurrence of the recurrent t(8;22)(q24;q11) translocation. **f**–**i** Clinical course of the patient depicted over a 4 months period with time points of CT scans performed at initial diagnosis (**f**), after a first chemotherapy cycle (**g**), and at disease progression (**h**), and different therapeutic interventions (**i**). **j, k** *TP53* deep targeted sequencing study of cells from the bulky mass and peritoneal fluid, showing the presence of R282W mutation in the peritoneal (ascitic) fluid cells but not in the bulky mass. **l** Sanger sequencing analysis confirming the presence of a heterozygous R282W mutation in the cells from peritoneal fluid, and lack of mutation in the bulky mass.

**Fig. 2:**
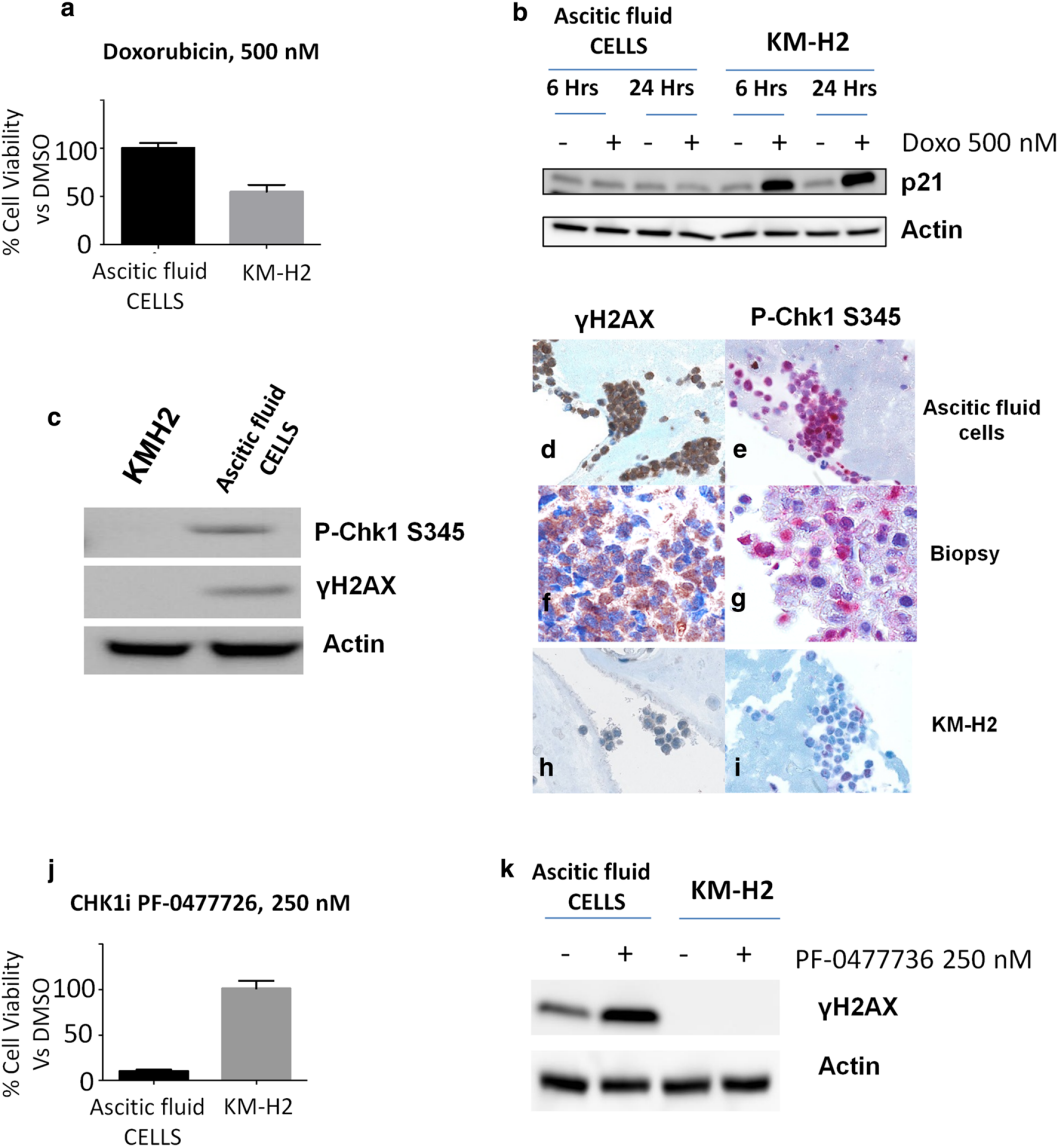
Functional ex vivo studies showing doxorubicin resistance and sensitivity to DDR inhibition in *TP53* mutant cells from peritoneal effusion.** a **WST-1 viability assay of primary ascitic fluid BL cells and KM-H2 cells treated with DMSO and doxorubicin 500 nM for 24 h. The percentage of viable cells after treatment in each cell line was normalized to its own untreated (DMSO) control.** b **Western blot analysis of BL *TP53* mutant primary cells and *TP53* wild type HL-derived KM-H2 cells showing p21 induction in KM-H2 cells after doxorubicin (doxo) treatment (500 nM for 6 and 24 h). **c** Western blot analysis of BL *TP53* mutant primary cells and *TP53* wild type HL-derived KM-H2 cells showing relative overexpression of pCHK1 S345 and pH2AX S139 in primary BL *TP53* mutant BL cells, compared to *TP53* wild type KM-H2 cells. **d**–**i** Immunocytochemistry for p-CHK1 S345, p-H2AX S139, in cultured primary cells from peritoneal fluid (**d**, **e**), the bulky mass (**f**, **g**), and KM-H2 cells (**g**, **h**) confirming western blot findings (×400). **j** WST-1 viability assay of primary BL cells from ascitic fluid and KM-H2 cells treated with DMSO and PF-0477736 250 nM for 24 h. The percentage of viable cells after treatment in each cell line was normalized to its own untreated (DMSO) control. **k** Western blot assay of *TP53* mutant primary BL cells from ascitic fluid and *TP53* wild type HL-derived KM-H2 cells showing pH2AX S139 induction in primary BL cells after PF-0477736 treatment (250 nM for 24 h).

The patient was hospitalized in August 2011 in critical conditions with two bulky abdominal masses originating from both ovaries, a massive abdominal effusion and small bowel obstruction. Surgical biopsy of the bulky mass (left ovary), cytology of the malignant cells from ascitic fluid, and immunophenotype (CD20+, CD19+, CD10+, BCL6+, CD38+, c-MYC) led to the diagnosis of BL (Fig. 1a–d). Fluorescence in situ hybridization (FISH) on malignant cells from both bulky mass and ascitic fluid showed a t(8;22)(q24;q11) translocation involving the *MYC* oncogene and the lambda light chain locus (IGL) (Fig. 1e). Detailed description of Immunohistochemistry and FISH studies is available in Additional file 1. The principal comorbidity was a severe bipolar disorder and anorexia nervosa that was still active at the time of disease onset, so that the patient was severely underweight (body mass index <17 kg/m^2^) and deemed initially unfit for intensive chemotherapy. Initial treatment consisted in 5 days of debulking cyclophosphamide (200 mg/m^2^/die) followed by 1 CHOP (cyclophosphamide, doxorubicin, vincristine, prednisone) cycle, which was complicated by severe tumor lysis syndrome, and bowel perforation requiring surgical intervention (Fig. 1f–i). A computed tomography (CT) scan performed after the first cycle showed marked reduction of the bulky lesions, with persistence of the abdominal effusion (Fig. 1g). After recovering from surgery, she received two additional Rituximab-CHOP-14 cycles but a CT scan performed right after showed marked disease progression (Fig. 1h). At this point the patient underwent therapy intensification according to the B-NHL-2002 regimen [12] but was unresponsive and ultimately died of rapidly progressing disease. Since recent studies confirmed that *TP53* mutations occur in about 30 % of BL cases [6, 7], in order to investigate the mechanisms underlying resistance to standard and intensive chemotherapy in this patient, we performed *TP53* targeted DNA sequencing of the tumor tissue available from the initial biopsy (left ovary), of tumor cells initially collected from the ascitic fluid, and of matched normal saliva using the 454 GS Junior platform (Roche diagnostics) (Additional file 1). The patient gave informed consent for the use of surplus tissue in research, and the protocol was approved by the Institutional Review Board (Study n 12/2009/U/Tess, protocol 148/2009). We found that the tumor tissue from the initial bulky mass was entirely *TP53* wild type, whereas lymphoma cells from the abdominal effusion harbored an heterozygous R282W mutation (Fig. 1j, k), which resulted in a 844C>T aminoacidic change, known to negatively affect p53 function and being associated to shorter survival in different cancer models (IARC database http://p53.iarc.fr/TP53GeneVariations.aspx) [13, 14]. These findings were confirmed by Sanger sequencing (Fig. 1l). Notably, both samples were chemonaive being collected before the start of chemotherapy. In order to define the impact of the R282W mutation on response to therapy in this specific case, we treated cultured *TP53* mutant primary BL cells from the ascitic fluid with either DMSO 0.01 % or doxorubicin 500 nM (Fig. 2a). The *TP53* wild type Hodgkin lymphoma cell line KM-H2 was used as a control. According to the *TP53* status, primary mutant BL cells were resistant to doxorubicin, whereas KM-H2 cells were sensitive. Consistent with these data, doxorubicin induced p21 expression in KM-H2 cells but not in primary BL cells (Fig. 2b). Interestingly, as shown in Fig. 1, while the *TP53* WT bulky masses rapidly responded to chemotherapy, the malignant *TP53* mutant ascites was still present at the time of second CT scan despite multiple repeated paracenteses (Fig. 1g), indicating a similar chemoresistant behavior also in vivo. Cell viability assays were performed by using WST-1 reagent (Roche). Detailed description of western blot protocols, antibodies and reagents is available in Additional file 1.

Since we recently reported constitutive DDR activation and high efficacy of CHK inhibitors in *TP53* mutant aggressive B-cell lymphomas (DLBCL and BL) [5], we evaluated the expression levels of genomic instability and DDR activation markers [5, 15] in peritoneal fluid cells and in the bulky mass by western blotting (Fig. 2c) and immunohistochemistry (Fig. 2d–i) confirming that peritoneal fluid cells demonstrated constitutive γH2AX (H2AX S139) and p-CHK1 S345 expression (Fig. 2c–e). Notably, although to a lesser extent, we observed positivity for these markers also in the *TP53* WT bulky mass (Fig. 2f, g), suggesting that the acquisition of genomic instability and of a DDR+ phenotype was an intrinsic feature of this neoplasm that preceded the development of the *TP53* mutation. The *TP53* WT KM-H2 cells, used as negative control of DDR activation [5], were negative for both p-CHK1 and γH2AX (Fig. 2h, i). Next, in order to assess whether the *TP53* mutant subclone was sensitive to DDR inhibition, we treated primary ascitic fluid BL cells (DDR+) and KM-H2 cells (DDR-) with the CHK inhibitor PF-0477736, finding that peritoneal fluid cells were exquisitely sensitive to CHK inhibition whereas KM-H2 cells were resistant (Fig. 2j). Following CHK inhibition, γH2AX levels increased in primary peritoneal fluid cells, indicating that in these cells the blockade of DDR leads to accumulation of endogenous DNA damage (Fig. 2k). These findings are consistent with a model in which constitutive activation of CHK kinases cooperates with MYC and is crucial to prevent untolerable levels of genomic instability deriving from *MYC*-induced replication stress and G1/S checkpoint dysfunction.

## Conclusions

These observations could have broad implications in clinical practice, suggesting that multiple tumor samples from different regions should be evaluated before tailoring therapies based on genome sequencing results. Although no definitive conclusions can be drawn from single case studies, this report strongly corroborates previous findings from our group and others showing efficacy of CHK inhibitors in *MYC*-driven and *TP53* mutant lymphoma models, suggesting that: (1) the occurrence of clonal heterogeneity at disease onset for mutations driving chemoresistance, such as those in *TP53*, should be taken into account in aggressive *MYC*-driven lymphomas; (2) CHK inhibitors could be effective in targeting hidden *TP53* mutant clones in tumors characterized by genomic instability and prone to intra-tumor heterogeneity. In conclusion, these data indicate that multiregion sequencing will be a crucial step for the development of precision therapy in aggressive B-cell lymphomas and confirm that inhibition of CHK kinases could be a suitable therapeutic strategy for *MYC*-driven tumors, which should be evaluated in future clinical trials.

## Consent

Written informed consent was obtained from the patient for publication of this case report and any accompanying images. A copy of the written consent is available for review by the Editor-in-Chief of this journal.

## Additional file

Additional file 1. Supplementary methods.

## Abbreviations

DDR: DNA damage response
BL: Burkitt lymphoma
DLBCL: diffuse large B cell lymphoma
CHK: checkpoint kinase
NGS: next generation sequencing
WT: wild type
FISH: fluorescence in situ hybridization
IGL: immunoglobulin lambda light chain locus
DMSO: dimethyl sulfoxide
