# Supplementary material for: Therapeutic implications of intratumor heterogeneity for TP53 mutational status in Burkitt lymphoma
Source: Exp Hematol Oncol. 2015 Aug 27;4:24. doi: 10.1186/s40164-015-0019-9 (PMC4549912; doi:10.1186/s40164-015-0019-9)
Supplement: Additional file 1. — Supplementary methods. [file 40164_2015_19_MOESM1_ESM.docx]

**Supplementary informations**

**Sample preparation, TP53 targeted sequencing**

A total of 1x10^9^ lymphoma cells were harvested during an evacuative paracentesis performed at disease onset. Mutational analysis of TP53 was done by next generation sequencing using the 454 GS Junior platform with 454 GS Junior Titanium chemistry for amplicon sequencing (Roche Diagnostics) and following the protocol developed in the IRON-II consortium. Ninety-sixe well plates containing dried down primers for the PCR amplification of *TP53* exons 4-11 in 11 samples were provided by IRON (Interlaboratory Robustness Of Next generation sequencing)-II study and used^1^. Primers for each sample were labeled with Multiplex Identifiers (MIDs). For each sample 320 ng of DNA, at a concentration of 20 ng/μl, were amplified by FastStart High Fidelity (Roche Diagnostics). PCR products were purified with the Agencourt AMPure XP Kit (Beckman Coulter, Krefeld, Germany) and quantified with the Quant-iT PicoGreen Assay (Life Technologies) before being pooled for each sample. Each amplicon pool was purified again with the Agencourt AMPure XP Kit and processed in a single NGS run. The subsequent 454 emulsion PCR amplification was carried out according to the manufacturer’s instructions and with a 0.4 ratio of DNA copies to beads. NGS data were analyzed by the Roche Diagnostics GS Run Browser and GS Amplicon Variant Analyzer software (version 2.7; Roche Diagnostics). Both forward and reverse reads were generated to validate the detected variants.

Mutations were compared with published single nucleotide polymorphism and mutations data, available through the Ensembl genome browser ([www.ensembl.org](http://www.ensembl.org)), dbSNP Database (<http://www.ncbi.nlm.nih.gov/SNP/>) and IARC TP53 Database (http://p53.iarc.fr/).

The sequencing data were analyzed by the Roche Diagnostics GS Run Browser and GS Amplicon Variant Analyzer software.

**TP53 Sanger sequencing.**

Total cellular RNA was extracted using the RNeasy total RNA isolation kit (Qiagen, Valencia, CA). One microgram of total RNA was reverse transcribed using the M-MLV Reverse Transcriptase (Invitrogen, San Diego, CA). Three overlapping shorter amplicons [amplicon 1 (491 bp): exons 1-5; amplicon 2 (482 bp): exons 5-8; amplicon 3 (498 bp): exons 8-11)] covering the entire coding sequence (GenBank accession number NM_000546.4) were amplified with 2U of FastStart Taq DNA Polymerase (Roche Diagnostics, Mannheim, Germany), 0.8 mM dNTPs, 1 mM MgCl2, and 0.2 M forward and reverse primers (Table S2) in 25 μl reaction volumes. PCR products were purified using QIAquick PCR purification kit (Qiagen) and then directly sequenced using an ABI PRISM 3730 automated DNA sequencer (Applied Biosystems, Foster City, CA) and a Big Dye Terminator DNA sequencing kit (Applied Biosystems). All sequence variations were detected by comparison using the BLAST software tool (www.ncbi.nlm.nih.gov/BLAST/) to reference genome sequence data (GenBank accession number NM_000546.4).

Primer sequences. For each primer pair, the sequence (5’- 3’), the melting temperature (Tm), the length and the amplicon size are reported. Primers have been designed using Primer3 (v. 0.4.0) Software Tool. P53 F1 [(TGGATTGGCAGCCAGACT), Temperature (T) 60.36 C°, Length 18, Amplicon size 491 base pair (bp)], P53 R1 [(GGGGGTGTGGAATCAACC), T 61.01 C°, length 18, amplicon size 491 bp], P53 F2 [(TCAACAAGATGTTTTGCCAACT), T 59.50 C°, length 22, amplicon size 482 bp], P53 R2 [(GCGGAGATTCTCTTCCTCTGT), T 59.97 C°, length 21, amplicon size 482 bp], P53 F3 [(GGTAATCTACTGGGACGGAACA), T 60.24 C°, length 22, amplicon size 498 bp], P53 R3 [(CTATTGCAAGCAAGGGTTCAA), T 60.25 C°, length 21, amplicon size 498 bp].

**Cell lines, and reagents**

The Hodgkin Lymphoma (HL) derived KM-H2 cells were obtained from the German Collection of Microorganisms and Cell Cultures, Department of Human and Animal Cell Cultures (Braunschweig, Germany). Cell lines were cultured in RPMI 1640 medium supplemented with 10% heat-inactivated fetal bovine serum (GIBCO BRL, Gaithersburg, MD), 1% l-glutamine, and penicillin-streptomycin in a humid environment of 5% CO_2_ at 37°C. Doxorubicin and PF-0477736 were purchased from SIGMA chemicals (Milan, Italy). The KM-H2 cell line was confirmed to be TP53 wild-type by Sanger sequencing.

**Immunohistochemistry**

Sections obtained from formalin-fixed paraffin embedded tissue of the ovary mass and of cytoinclusion of the ascitic fluid (cells centrifuged, washed in PBS, fixed in 10% buffered formalin and then included in paraffin as reported previously)^2^ were investigated by antibodies raised against fixation resistant epitopes of p-CHK1 serine (ser) 345 (rabbit polyclonal, dilution 1:20, Novus Biologicals: NBPI-60799), p-H2AX ser 319 (rabbit polyclonal, dilution 1:25, Cell Signaling:2577), CD20 (mouse monoclonal, 1:200, Dako: M0755), CD19 (mouse monoclonal, dilution 1:200, Dako: M7296), CD10 (mouse monoclonal, 1:40, Leica: ORG8941), BCL6 (mouse monoclonal, undiluted, clone PGb6p, provided Prof Falini, Perugia), CD38 (mouse monoclonal,1:20, Novocastra: NCL-L_CD38-290), c-MYC (rabbit monoclonal, dilution 1:100, Epitomics: 1472)

The antibody reactivity as well as the antigen retrieval protocols and revelation systems were previously reported^3^. Immunohistochemical preparations were visualized and images were captured using Olympus Dot-slide microscope digital system equipped with the VS110 image analysis software.

**Fluorescence In Situ Hybridization** (FISH)

Chromosome preparations were hybridized in situ with 1μg of each probe labeled by nick translation. The whole chromosome paints used for chromosomes 8 and 22, derived from flow sorted chromosomes, were a gift of the Sanger Center (Dr Nigel Carter). Hybridization was performed at 37°C in 2X saline sodium citrate, 50% (vol/vol) formamide, 10% (wt/vol) dextran sulfate, 5μg of COT1 DNA (Bethesda Research Laboratories, Gaithersburg, MD, USA), and 3μg of sonicated salmon sperm DNAin a volume of 10 μL. Post-hybridization washing was performed at 60°C in 0.1X saline sodium citrate (3 times). In cohybridization experiments, the probes were directly labeled with FITC (green; Fermentas Life Sciences, Milan, IT), and Cy3 (red; New England Nuclear, Boston, MA, USA). Chromosomes were identified by DAPI (blue) staining. Digital images were obtained by the use of a Leica DMRXA epifluorescence microscope equipped with a cooled CCD camera (Princeton Instruments, Boston, MA). Cy3, FITC and DAPI fluorescence signals, which were detected by the use of specific filters, were recorded separately as gray-scale images. Pseudocoloring and merging of images were performed with Adobe Photoshop software.

**Western blotting**

A total of 30 μg of protein was denatured in Laemmli buffer at 95°C and separated by SDS-PAGE. Proteins were then electrotransferred onto nitrocellulose membranes and submitted to immunodetection with the relevant antibody. Membrane-bound secondary antibodies (HRP-conjugated goat anti-rabbit or anti-mouse, BioRad) were detected using SuperSignal West Dura Extended Duration Substrate (Pierce Chemical Co., Rockford, IL). For western blotting, antibodies to the following were purchased from Cell Signaling Technology: p-CHK1 ser 345, p-H2AX ser 139, p21, Actin.

**References**

1. Kohlmann A, Martinelli G, Hofmann W-K, et al. The Interlaboratory Robustness Of Next-Generation Sequencing (IRON) Study Phase II: Deep-Sequencing Analyses Of Hematological Malignancies Performed In 8,867 Cases By An International Network Involving 27 Laboratories. Blood, 2013;122, 21, Abstr 743.
2. Agostinelli C, Paterson JC, Gupta R, et al. Detection of LIM domain only 2 (LMO2) in normal human tissues and haematopoietic and non-haematopoietic tumours using a newly developed rabbit monoclonal antibody. Histopathology. 2012; 61(1):33-46.
3. Derenzini E, Agostinelli C, Imbrogno E et al. Constitutive activation of the DNA damage response pathway as a novel therapeutic target in diffuse large B-cell lymphoma. Oncotarget. 2015, 6(9):6553-69.
